# Integrating 16S rRNA Sequencing and LC-MS-Based Metabolomics to Evaluate the Effects of Dietary Crude Protein on Ruminal Morphology, Fermentation Parameter and Digestive Enzyme Activity in Tibetan Sheep

**DOI:** 10.3390/ani14152149

**Published:** 2024-07-24

**Authors:** Zhenling Wu, Fengshuo Zhang, Quyangangmao Su, Qiurong Ji, Kaina Zhu, Yu Zhang, Shengzhen Hou, Linsheng Gui

**Affiliations:** College of Agriculture and Animal Husbandry, Qinghai University, Xining 810016, China; w15165514762@163.com (Z.W.); zhangfengshuo1997@163.com (F.Z.); qyam2737@163.com (Q.S.); jqr1960742393@163.com (Q.J.); 15291938937@163.com (K.Z.); iszhangyu06@163.com (Y.Z.); qhdxhsz@163.com (S.H.)

**Keywords:** protein level, Tibetan sheep, rumen development, microbiota, metabolites

## Abstract

**Simple Summary:**

Dietary protein satisfies growth performance requirements and contributes to the maintenance of the rumen environment. High protein intake increased the total volatile fatty acids (VFAs), propionic acids and butyric acids by regulating the microbiome (*Prevotella 1*, *Rikenellaceae RC9 gut group*, *Candidatus Saccharimonas*, *Ruminococcus 1*, *Coprostanoligenes group*, *Ruminococcaceae UCG-014*, *Lachnospiraceae NK3A20 group* and *Succiniclasticum*) and metabolome (stearic acid, oleic acid, palmitic acid, erucic acid, 6-hydroxymelatonin, indole-3-acetamide and taurine), thereby altering the papillae length and papillae width in the rumen of Tibetan sheep.

**Abstract:**

The dietary crude protein level could affect ruminal fermentation parameters and the microflora of ruminants. The present study’s aim was to investigate the effects of different protein level diets on ruminal morphology, fermentation parameters, digestive enzyme activity, microflora and metabolites of Tibetan sheep. Ninety weaned lambs (initial weight of 15.40 ± 0.81 kg, 2 months old) were selected and randomly divided into three groups (six pens/treatment, five rams/pen). Dietary treatments were formulated with 13.03% (high protein, HP), 11.58% (moderate protein, MP) and 10.20% (low protein, LP), respectively. Compared with LP, both papillae length and papillae width were significantly promoted in HP and MP (*p* < 0.05). The concentrations of ammonia nitrogen, total VFAs, propionic acids and butyric acids in HP were significantly increased compared to those in MP and LP (*p* < 0.05). The activities of protease and α-amylase in HP were significantly greater than those of LP (*p* < 0.05). For the ruminal microbial community, higher proportions of phylum *Prevotella 1* and *Succiniclasticum* and genus *Rikenellaceae RC9 gut group* and *Ruminococcus 1* were observed in HP (*p* < 0.05). A total of 60 differential metabolites (DMs) (28 up, 32 down) between HP and MP; 73 DMs (55 up, 18 down) between HP and LP; and 65 DMs (49 up, 16 down) between MP and LP were identified. Furthermore, four pathways of the biosynthesis of unsaturated fatty acids, tryptophan metabolism, bile secretion and ABC transporters were significantly different (*p* < 0.05). The abundance of phylum *Prevotella 1* was negatively associated with stearic acid and palmitic acid but positively associated with the taurine. The abundance of genus *Ruminococcus 1* was negatively associated with stearic acid, oleic acid, erucic acid, Indole-3-acetamide and palmitic acid but positively associated with 6-hydroxymelatonin. In conclusion, a 13.03% CP level improved ruminal morphology, fermentation parameters and digestive enzyme activities through modulating the microbial community and regulating metabolism in Tibetan sheep.

## 1. Introduction

Tibetan sheep (*Ovis aries*) are one of the most economically important domestic animals on the Qinghai–Tibetan Plateau. They are well adapted to the high-altitude and low-oxygen environment and provide a basic livelihood for local herders [1]. In addition, Tibetan sheep play an important role in maintaining the ecological balance of the Qinghai–Tibetan Plateau [2]. The long cold season from November to May each year, with average temperatures ranging from −5 °C to 15 °C, has resulted in extreme shortages of grass productivity and pasture nutrients (especially crude protein content) [3]. Therefore, supplementing an appropriate amount of protein in their diet is an effective strategy for alleviating the pressure on protein feed supply during standardized breeding and to improve the growth performance of Tibetan sheep.

The rumen is the primary organ system for nutrient digestion and absorption in ruminants, and it contains abundant microbiota and metabolites [4]. Plant fibers and polysaccharides in feed can be degraded into VFAs by rumen microorganisms, which are the main source of energy for the body and can stimulate the development of rumen epithelial tissue [5]. Ration composition and nutrient content are important factors influencing the microbiota and metabolites; therefore, a comprehensive analysis of rumen fluid can provide insights into the interaction between rations and the rumen in ruminants [6,7]. The study of rumen microorganisms and metabolites in yaks showed that the level of protein in their diet could significantly affect the composition of rumen microbial flora and cause changes in the metabolic pathways of metabolites, also affecting the rumen fermentation index and rumen epithelial tissue development [8]. A study on the rumen microflora and metabolites of cashmere goats showed that a high-energy/high-protein diet could significantly change the structure of the rumen microflora and the composition of metabolites, improve the utilization efficiency of energy and protein, and significantly alter metabolites [9].

Currently, few studies have applied a combined meta-omics approach to evaluate the effects of dietary protein levels on the ruminal function of Tibetan sheep. Previous research suggested that a high dietary protein level promoted feed intake and nutrient digestibility. Therefore, it is hypothesized that an appropriate dietary protein level could affect the ruminal function of Tibetan sheep. The present study aimed to investigate the effects of different protein level diets on ruminal morphology, fermentation parameters, digestive enzyme activities, microflora and metabolites of Tibetan sheep.

## 2. Materials and Methods

Animal care and experimental protocols were approved (QUA-2020-0710) by the Institutional Animal Care and Use Committee of the Qinghai University, China.

### 2.1. Animal and Experimental Design

A total of 90 2-month-old male weaned lambs (initial weight of 15.40 ± 0.81 kg) were purchased from Jingzang Animal Husbandry Co., Ltd. (Haiyan, China), which were randomly divided into three groups (6 pens/treatment, 5 rams/pen). Dietary treatments were formulated with 13.03% (HP), 11.58% (MP) and 10.20% (LP), respectively. The test period lasted 97 days, consisting of 7 days for adaptations. This experiment was carried out using a single-factor randomized block design. The diets consisted of 30% forage and 70% concentration on a dry matter basis. The ingredients and chemical compositions of diets are shown in Table 1. All lambs were provided adequate water levels and were fed twice a day at 08:00 and 17:00.

The chemical composition of the ingredients, including crude protein, ether extract, acid detergent fiber, neutral detergent fiber, Ca and P, were measured according to a method described previously [10].

### 2.2. Sample Collection

At the end of the formal trial, 18 sheep (*n* = 6 per treatment) were randomly selected and slaughtered at a commercial slaughterhouse. To standardize the sample collection process across samples, samples were mainly collected from the center of the lateral rumen wall. The rumen fluids were collected under aseptic conditions, filtered through four-layered sterilized gauze and placed in sterile enzyme-free freezing tubes (ThermoFisher, Waltham, MA, USA). All samples were stored in liquid nitrogen until future analysis.

### 2.3. Rumen Epithelial Morphology

The ruminal papilla length and papilla width were observed under a microscope following H&E staining. Briefly, the tissue samples were fixed in 4% paraformaldehyde for no less than 48 h, dehydrated in serial ethanol concentrations and embedded in paraffin. The tissue samples were then cut into thin 5 μm sections and mounted on glass slides. Finally, the tissues were observed and images were captured using Image Pro Plus 5.1 (Media Cybernetics Inc., Bethesda, MD, USA) to determine different rumen tissue indicators.

### 2.4. Fermentation Parameters

The pH of the rumen fluid was determined using the convenient acidimeter (Sangon, Shanghai, China). The concentrations of VFAs were measured using gas chromatography (GC-2010, Agilent, Kyoto, Japan). Briefly, the rumen fluids were filtered by four layered sieves and then mixed with 25% metaphosphate (5:1). The mixture was centrifuged at 10,000× rpm for 10 min and the supernatant was collected. The concentration of VFAs in the samples was measured under DB-FFAP capillary column (30 m × 0.32 mm × 0.25 μm). The ammonia nitrogen contents of rumen fluid were determined using the phenol sodium hypochlorite colorimetric method [11].

### 2.5. Digestive Enzyme Activities

The activities of the digestive enzymes, including protease, α-amylase, cellulase and lipase, were determined using the enzyme-linked immunosorbent assay kit (Meibiao Biotechnology Co., Ltd., Yancheng, China).

### 2.6. Microbiome Composition Analysis

DNA was extracted using the HiPure Stool DNA extraction kit (Magen, Guangzhou, China). The primers used for the PCR process were 515 F (5′-CCTAYGGGRBGCASCAG-3′) and 806 R (5′-GTGCCAGCMGCCGCGG-3′) and were specific for the V3–V4 region of the bacterial 16S rRNA gene. The constructed library was quantified by NEB Next^®^Ultra™DNA Library Prep Kit for Illumina (Diego, CA, USA), and index codes were added. The library quality was assessed on the Qubit@ 2.0 Fluorometer (Carlsbad, CA, USA). Lastly, the DNA library was sequenced on an Illumina MiSeq platform, and 250 bp paired-end reads were generated.

To obtain the effective tags, FLASH software (version 0.18.0) was used to splice from the raw tags of the sequences. The effective tags were clustered into operational taxonomic units (OTUs) of ≥97% similarity using the Uparse pipeline [12]. Alpha diversity analyses were calculated in QIIME (version 1.9.1) [13]. The beta distance was calculated using the R language package Vegan (version 2.5.3). Bray–Curtis distance matrix dissimilarities were assessed using the R “ggplot2” package (version 2.2.1) [14].

### 2.7. Metabolomics Data Analysis

The rumen fluid blended with 1 mL of the precooled methanol acetonitrile water solution (methanol/acetonitrile/water = 2:2:1, *v*/*v*) and then centrifuged for 20 min at 10,000× rpm for LC-MS/MS analysis. The metabolites were separated by the Agilent 1290 Infinity LC ultra-high performance liquid chromatography system (UHPLC). Samples were placed in a 4 °C autosampler (25 °C column temperature). The gas flow rate through the column was 0.5 mL/min.

Primary and secondary spectra of the samples were acquired using the AB Triple TOF 6600 mass spectrometer, with the ESI conditions applied as described by Zhang et al. [15]. Before normalizing the total peak intensity, raw data (MzXML files) were first imported to the XCMS software (version 4.0). The processed data were uploaded before importing into SIMCA-P (version 14.1, Umetrics, Umea, Sweden). The data were subjected to multivariate analysis with orthogonal partial least square discriminant analysis (OPLS-DA), and significantly differential metabolites (DMs) were screened based on variable importance in projection (VIP) scores (VIP > 1) from OPLS-DA and *p*-values (*p* < 0.05). Finally, the Kyoto Encyclopedia of Genes and Genomes (KEGG, http://www.genome.jp/kegg/, accessed on 29 August 2022) and MetaboAnalyst3 databases were used for pathway enrichment analysis.

### 2.8. Statistical Analysis

Spearman correlation analysis was used to evaluate the relationship among metabolin, metabolin, ruminal morphology, fermentation parameters and digestive enzyme activity. 

All data were analyzed by a general linear model (GLM) using SPSS 25.0 software (IBM Corp., Armonk, NY, USA). The results were expressed as “mean ± standard error”, and *p* < 0.05 represented significant difference.

## 3. Results

### 3.1. Histological Analysis of the Rumen

**Figure 1:**
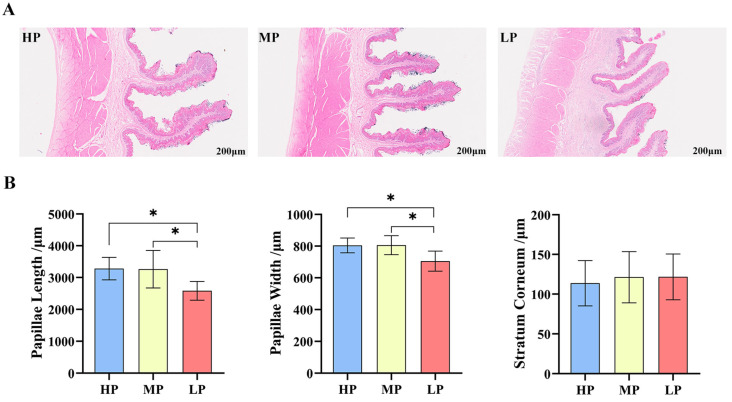
Effect of dietary protein level on ruminal phenotypes. (**A**) Representative histological images of ruminal slide stained with hematoxylin–eosin (original magnification 200× μm). (**B**) The papillae height, papillae width and stratum corneum of rumen. * *p* < 0.05.

H&E staining results of rumen papillae are shown in Figure 1A. Significant differences were observed in papillae length and papillae width among the three treatments. The papillae length and papillae width of HP and MP were significantly longer and wider, respectively, than those of LP (*p* < 0.05). No obvious difference was observed in the stratum corneum among the groups (*p* > 0.05) (Figure 1B).

### 3.2. Rumen Fermentation Parameter

The fermentation parameter results are shown in Table 2. No significant difference was observed in acetic acid, isobutyric acid and pH among the three groups (*p* > 0.05). Compared with the LP group, the ammonia nitrogen, total VFAs, propionic acid and butyric acid content were significantly higher than in the HP (*p* < 0.05).

### 3.3. The Activity of Digestive Enzymes in the Rumen

The activities of digestive enzymes, including trypsin, α-amylase, lipase and protease, were evaluated (Table 3). The activities of trypsin and α-amylase were significantly higher in HP than in LP (*p* < 0.05). However, no significant difference was observed in lipase and protease activities (*p* > 0.05).

### 3.4. Bacterial Community

**Figure 2:**
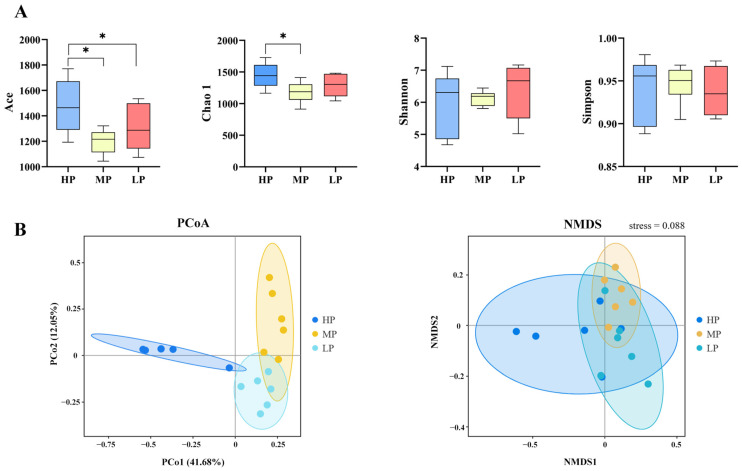
Effect of dietary protein level on bacterial community. (**A**) Alpha diversity analysis. (**B**) Beta diversity analysis. * *p* < 0.05.

A total of 4092 OTUs were identified across the three groups. Of these, 1079, 269 and 580 OTUs were identified in HP, MP and LP, respectively (Figure S1). Alpha diversity analysis revealed the diversity of bacteria in HP (Figure 2A). The Ace Chao1 were significantly higher in HP than in MP (*p* < 0.05). Principal co-ordinate analysis (PCoA) and non-metric multi-dimensional scaling (NMDS) showed clear separation of rumen samples from the three treatment groups (Figure 2B).

**Figure 3:**
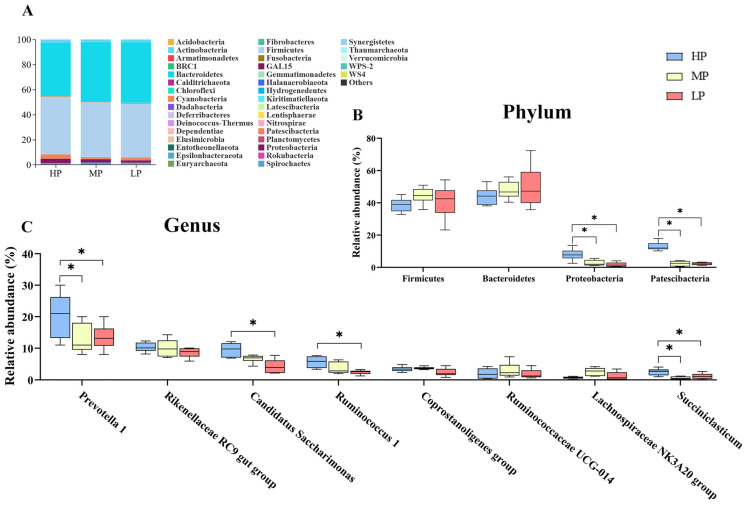
Effect of dietary protein level on bacterial composition. (**A**) Bacterial composition at the phylum. Significantly different bacterial phylum (**B**) and genus (**C**) between groups. * *p* < 0.05.

*Bacteroidetes* was the dominant rumen phyla across the three groups, which accounted for 42.53%, 47.78% and 48.26%, respectively, of bacteria in the HP, MP and LP groups (Figure 3A). No significant difference was observed in the relative abundance of *Firmicutes* and *Bacteroidetes* among the three groups (*p* > 0.05). However, the abundance of *Proteobacteria* and *Patescibacteria* was significantly higher in HP than in the other groups (*p* < 0.05) (Figure 3B). The top eight most abundant genera were also identified across the three groups (Figure 3C). Low dietary protein levels significantly decreased the abundance of *Prevotella 1*, *Candidatus Saccharimonas*, *Ruminococcus 1* and *Succiniclasticum* (*p* < 0.05).

### 3.5. Metabolomics Profiling of Rumen

**Figure 4:**
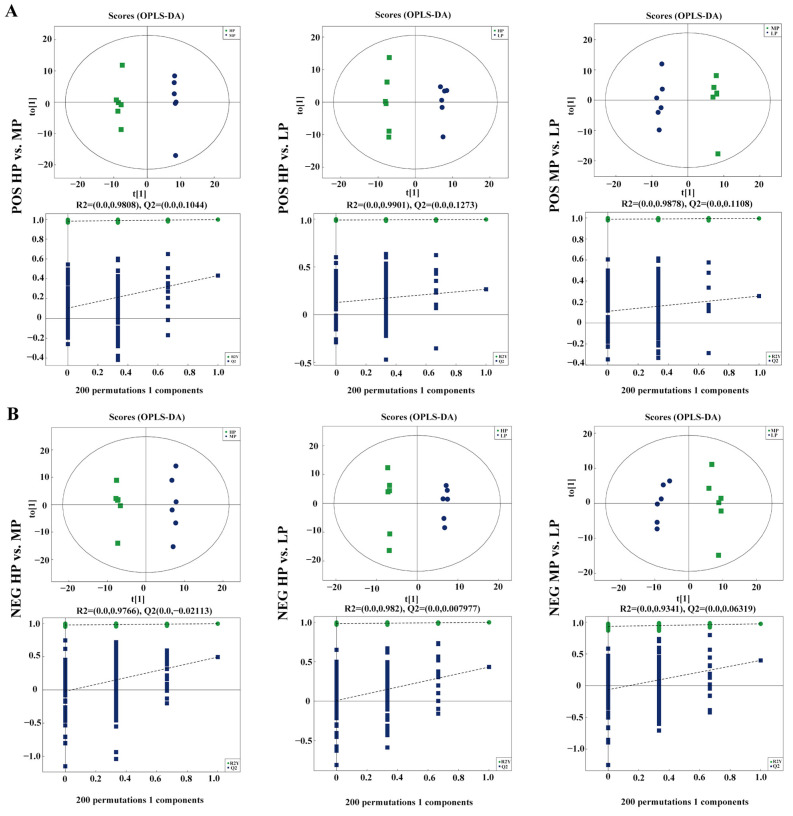
Orthogonal partial least squares discriminant analysis (OPLS-DA) plot of metabolites in comparisons. (**A**) Positive ion mode. (**B**) Negative ion mode.

The correlation coefficients between the QC samples were all above 0.9 (Figure S2), indicating that the data obtained in this experiment exhibited good reproducibility and precision. Further evaluation of the parameters of the OPLS-DA model showed that the ruminal fluids between groups exhibited significantly different metabolite compositions in positive ion (Figure 4A) and negative ion (Figure 4B), which indicated that metabolites obtained in the ruminal fluid samples of the three treatments were markedly distinct.

**Figure 5:**
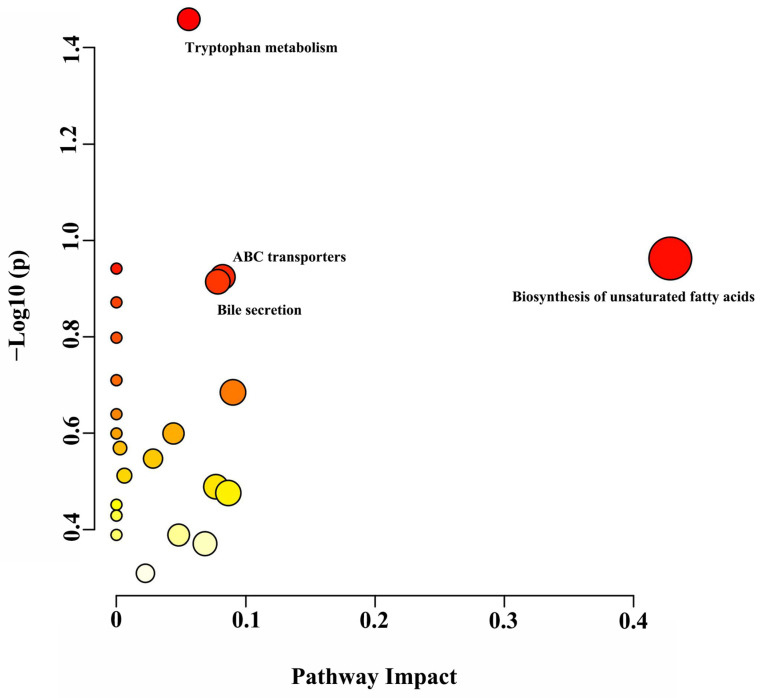
Metabolome view map of the differentially expressed metabolites identified. The deep color shows higher pathway impact values; the larger size demonstrates higher pathway enrichment. The important pathways selected for this experiment have been labelled in the figure.

A total of 1149 metabolites, which could be classified into “lipids and lipid-like molecules”, “organic acids and derivatives”, “organoheterocyclic compounds”, “benzenoids” and “organic oxygen compounds”, were identified (Figure S3). Based on the VIP (VIP > 1) and p-value (*p* < 0.05), differential metabolites among treatments were selected (Figure S4). Compared with the MP group, 60 differential metabolites (28 upregulated and 32 downregulated) were present in the HP group. Compared with the LP group, 73 differential metabolites (55 upregulated and 18 downregulated) were identified in the HP group. Compared with the LP group, 65 differential metabolites (49 upregulated and 16 downregulated) were identified in the MP. Four pathways, including biosynthesis of unsaturated fatty acids, tryptophan metabolism, bile secretion and ABC transporter pathways, were significantly different (Figure 5). Several differential metabolites, including stearic acid, oleic acid, palmitic acid, erucic acid, 6-hydroxymelatonin, indole-3-acetamide and taurine, were mapped to significantly different pathways.

### 3.6. Correlation Analysis

**Figure 6:**
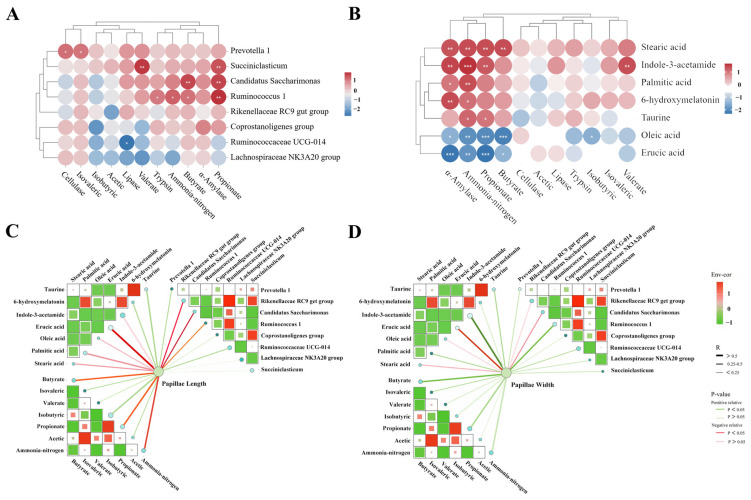
Spearman correlation analysis. (**A**) Association analysis of microbial diversity with rumen fermentation parameters and digestive enzyme activity. (**B**) Association analysis of rumen metabolites with rumen fermentation parameters and digestive enzyme activity. (**C**) Association analysis of rumen papillae length with rumen fermentation parameters, digestive enzyme activity and microbial diversity. (**D**) Association analysis of rumen papillae width with rumen fermentation parameters, digestive enzyme activity and microbial diversity. The color intensity of the circle and line are proportional to the correlation values. * *p* < 0.05, ** 0.05 > *p* < 0.01, *** *p* < 0.01.

The correlation network analysis was performed to assess the association between ruminal morphology, digestive enzyme activity, fermentation parameters, metabolites and the diversity/abundance/richness of microbiota. The abundances of *Succiniclasticum*, *Candidatus Saccharimonas* and *Ruminococcus 1* were positively correlated with propionate content. In addition, the abundances of *Candidatus Saccharimonas* and *Ruminococcus 1* were positively correlated with butyrate content (Figure 6A). As shown in Figure 6B, the propionate content was positively correlated with stearic acid, indole-3-acetamide, taurine and oleic acid content but was negatively correlated with erucic acid content. The butyrate content was positively correlated with stearic and oleic acid content but negatively correlated with erucic acid content. The papillae length was positively correlated with *Ruminococcaceae UCG-014*, *Lachnospiraceae NK3A20 group*, *Succiniclasticum* and *Rikenellaceae RC9 gut group* and *Candidatus Saccharimonas* (Figure 6C). The papillae width was positively correlated with *Ruminococcus 1*, indole-3-acetamide and butyrate (Figure 6D).

**Figure 7:**
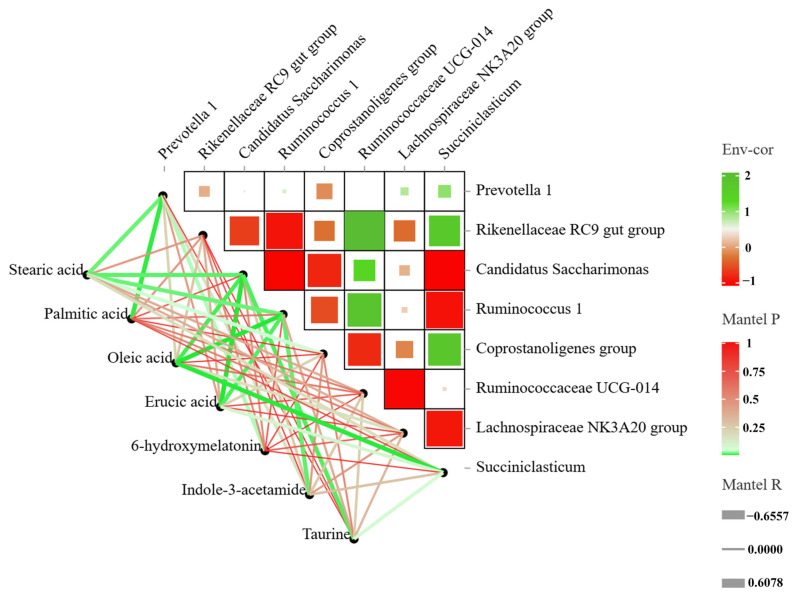
The Mantel correlation analysis indicated the correlation between rumen microorganisms and metabolites. The edge width corresponds to the distance correlation corresponding to the Mantel’s r statistic, and the edge color indicates statistical significance.

Mantel correlation analysis between the differential microbiome and the differential metabolite was also analyzed (Figure 7). The abundance of *Prevotella 1* was negatively correlated with stearic acid and palmitic acid. The abundance of *Rikenellaceae RC9 gut group* was positively correlated with indole-3-acetamide and taurine content. The abundance of *Candidatus Saccharimonas* was negatively correlated with stearic acid, oleic acid, erucic acid and taurine content but it was also negatively correlated with palmitic acid content. The abundance of *Ruminococcus 1* was negatively correlated with stearic acid, oleic acid, erucic acid and indole-3-acetamide contents.

## 4. Discussion

Dietary protein constitutes a vital source of nutrition for ruminants [3]. It not only provides growth requirements but also contributes to the maintenance of a healthy rumen environment [16,17]. Studies have shown that appropriate ammonia nitrogen levels are crucial for efficient microbial protein synthesis [18]. Zhu et al. reported that a low-protein diet decreased ammonia nitrogen concentration, consistent with the findings of the present study [19]. In the present study, a positive correlation was found between the protein content and ammonia nitrogen concentration. An appropriate rumen pH value (6.2–7.0) ensures optimal permeability of rumen microbial cell membranes and the density of rumen microbiota is achieved, thereby maintaining the stability of the rumen environment [20]. In the present study, no significant difference in pH was observed among the three groups. Regulation of rumen pH in ruminants is a complex process. It involves various factors that influence the production of saliva and VFAs, as well as microbial nitrogen, all of which contribute to the rumen pH stability [21,22]. The fattening period of Tibetan sheep is characterized by rapid growth and development, during which the rumen is mature and has strong fermentation ability, and the ruminant requires a high energy supply [23]. The concentration of total VFAs in the rumen of Tibetan sheep increased with protein levels. High levels of VFAs ensure efficient production performance of Tibetan sheep and enhance their adaptability to high altitude and cold environments [23,24]. In the present study, the concentration of total VFAs was higher, which may have contributed to the lower pH in the rumen of the high-protein group.

VFAs serve not only as a source of energy for the host animal but also influence the development of rumen epithelial tissue [25,26]. Propionic acid can act as a signaling molecule to stimulate mRNA expression for *GPR41* and *GPR43* in the rumen epithelium [27]. Additionally, butyric acid can activate the Monocarboxylate Transporter 1 (MCT1) promoter region in Caco-2 and HT-29 cell lines, thereby enhancing the expression of MCT1 mRNA and facilitating rumen development [28]. The present study revealed that the concentrations of propionic and butyric acids were significantly higher in the rumen of the high-protein group than in the low-protein group. The morphology of rumen tissue revealed that the dietary protein levels significantly affected the length and width of rumen papillae. The high-protein group exhibited significantly longer length and wider width of rumen papillae than the low-protein group. Consequently, it can be inferred that higher dietary protein promotes the synthesis of more short-chain fatty acids (SCFAs) in the Tibetan sheep rumen. In effect, this better sustains the energy needs of the rumen epithelium and enhances the expression of rumen epithelial-related genes, thereby promoting rumen development.

Enzymatic activity in the rumen fluid is closely related to the quantity and rumen microorganism-related metabolism [29,30]. The activities of cellulase, protease, α-amylase and lipase reflect the number and vigor of catabolic bacteria in the rumen [31,32]. In this study, it was found that protease activity was higher in the high-protein group than in the medium- and low-protein groups. This suggests that high-protein diets significantly increase protease activity, and this may be attributed to the higher level of ammoniacal nitrogen content in the rumen of the high-protein group, which provides a nitrogen source that promotes the synthesis of proteases by protein-degrading bacteria [33,34]. No significant difference was observed in the lipase and cellulase activities among the three groups in the present study. In a previous study, it was hypothesized that the increase in the activities of lipase and cellulase could be related to the fat content in the feed groups and the substrate specificity of digestive enzymes [35]. Additionally, the abundance of rumen microorganisms was not sufficient enough to cause significant changes in the degradation of crude fiber, which explains the indifference in cellulase activity among the three groups [36]. Furthermore, α-amylase activity was significantly higher in the high-protein group than in the low-protein group, and this may be attributed to the proliferation of microorganisms in the rumen at high protein levels and the high energy demand for starch degradation, leading to an increase in the α-amylase secretion by starch-degrading bacteria.

In this study, the Ace and Chao1 indices were significantly higher in the high-protein group than in the low-protein group, and the Ace index was also significantly higher in the high-protein group than in the medium-protein group. Collectively, the findings of the present study show that higher dietary protein content increases the abundance of rumen flora. Dai et al. reported similar findings in yaks [3]. In the present study, it was found that *Bacteroidetes* and *Firmicutes* were the most abundant bacteria in the rumen, consistent with previous findings [37,38]. The greater the bacteria abundance, the more beneficial it is for the rumen to utilize nutrients, which is crucial for rumen metabolism in ruminants [39]. At the genus level, *Prevotella 1* was the most abundant. The relative abundance of *Prevotella 1* was significantly higher in the high-protein group than in the medium- and low-protein groups, consistent with a previous finding [40]. *Prevotella 1* is not only involved in starch and protein utilization but also in the degradation of hemicellulose and pectin [41,42]. This may explain the significant positive correlation between the abundance of *Prevotella 1* and cellulose activity.

Additionally, it has been demonstrated that isobutyric acid promotes the growth of fiber-degrading bacteria in the rumen. This explains the positive correlation between isobutyric and isovaleric acids and the abundance of *Prevotella 1* [43,44]. *Succiniclasticum* plays a crucial role in maintaining rumen structure and function, as well as regulating the homeostasis of the ruminal internal environment. It also participates in the production of acetic and succinic acids [45]. In the present study, it was found that the abundance of *Succiniclasticum* was significantly higher in the high-protein group than in the medium- and low-protein groups. Additionally, there was a strong positive correlation between the abundance of *Candidatus Saccharimonas* and propionic acid as well as butyric acid contents, consistent with the findings of Wang et al. [46]. This indicates that *Candidatus Saccharimonas* participates in propionic and butyric acid metabolism. *Ruminococcus 1* participates in the decomposition of cellulose and starch. Members in this genus secrete abundant propionic and acetic acids [47,48]. In the present study, the abundance of *Ruminococcus 1* strongly and positively correlated with protease, propionic acid, butyric acid and ammonia nitrogen contents. Therefore, a high-protein diet can provide more fermentation substrates for *Ruminococcus 1*, leading to an increase in microbial population and, consequently, enhancing the efficiency of nutrient digestion [49].

To delve deeper into the impact of varying protein levels on rumen metabolites, metabolomics was used to determine the alterations in the content of rumen metabolites. The results showed that dietary protein levels have a significant impact on rumen metabolites. The main metabolites in the rumen (e.g., lipids and lipid-like molecules, organoheterocyclic compounds, organic oxygen compounds, etc.) are produced by the digestion of feeds by rumen microbial flora [50]. Meanwhile, dietary nutrients can alter the characteristics of rumen metabolomics in Tibetan sheep [51]. Studies have shown that unsaturated fatty acids (UFAs) exhibit mild toxicity to rumen microorganisms, and rumen hydrogenation alleviates this toxicity [52]. Metabolites in the unsaturated fatty acid biosynthesis synthesis pathway, including stearic acid, oleic acid, palmitic acid, and erucic acid, were selected for analysis. It was found that feed type significantly impacted the synthesis and metabolism of UFAs in the rumen. The high-protein group had significantly higher stearic and palmitic acids than the medium- and low-protein groups. Conversely, oleic and erucic acids were significantly lower in the high-protein group than in the other two groups. In conclusion, medium- and low-protein diets disrupt the breakdown of unsaturated fatty acid double bonds by rumen microorganisms, thereby impairing biohydrogenation in the rumen [53]. Combined with rumen microbiome analysis, this finding is more conducive to VFA production and increasing ammonia nitrogen concentration. This also explains the strong negative correlation between erucic and oleic acid contents and α-amylase activity, ammonia nitrogen, propionic acid and butyric acid concentration. Conversely, there was a strong correlation between stearic acid content and amylase activity, ammonia nitrogen, propionic acid and butyric acid concentration. Oleic acid (UFAs), a product of lipid metabolism, is hydrogenated and detoxified by microorganisms in the rumen to produce stearic acid (SFAs), which further supports the hypothesis of the present study [54]. The positive correlation between palmitic acid content and ammonia nitrogen concentration is attributed to palmitic acid’s ability to enhance rumen fermentation in ruminants [55]. Studies have shown that 6-hydroxymelatonin can regulate gastrointestinal peristalsis and promote gastrointestinal development, while Indole-3-acetamide plays an important role in regulating intestinal homeostasis [56,57]. Notably, a strong positive correlation was found between Indole-3-acetamide and acetic acid concentrations. It is hypothesized that Indole-3-acetamide promotes the growth of acetic-acid-producing/degrading bacteria in the rumen, and the specific mechanism of action requires further exploration. Bile, a component of animal digestive fluids, plays a crucial role in maintaining a healthy gut microbiota and regulating animal lipid metabolism and immunity [58]. In this study, the bile was significantly upregulated, suggesting that a high-protein diet promotes bile secretion and enhances lipid digestion and absorption. As a metabolite of ABC transporters, taurine regulates the production of hydrogen in the rumen. This regulation enhances rumen fermentation, improves rumen microbial crude protein synthesis and enhances fiber digestibility [59,60].

**Figure 8:**
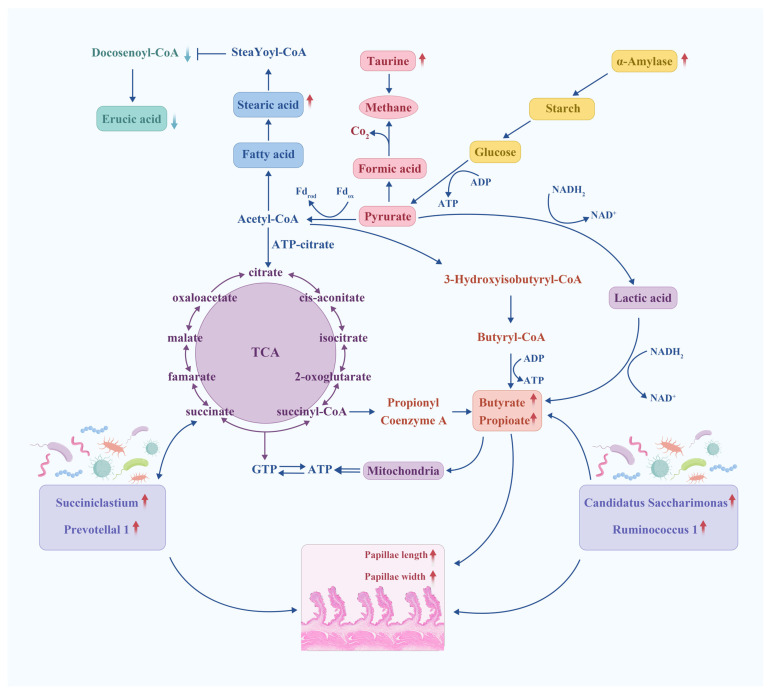
The mechanism diagram shows that the composition of rumen microbiota, rumen metabolites, fermentation parameters and digestive enzyme activity directly or indirectly alter rumen morphology at high protein levels. Red arrows indicate significant increases; green arrows indicate significant decreases.

The development of rumen papillae is influenced by the interaction among rumen microbiota, rumen metabolic substrates and the host, which varies with diet composition [61,62]. A study by Yin et al. [63] revealed that the abundance of *Ruminococcaceae UCG-014* and Lachnospiraceae NK3A20 was strongly and positively correlated with nipple length and width, while that of *Rikenellaceae RC9 gut group* was strongly and negatively correlated with both nipple length and width. These findings are both consistent with the findings of the present study. The *Lachnospiraceae NK3A20 group* constitutes a significant proportion of the rumen microbiota and is closely associated with the concentration of butyrate in the rumen [64,65]. The infusion of butyric acid increases the length, width, and surface area of rumen papillae in cattle [66]. *Rikenellaceae RC9 gut group* belongs to the *Rikenellaceae RC9* family, and the cellulose level in the rumen is linked to the activity of the *Rikenellaceae RC9 gut group* [67]. Combined with rumen cellulase activity in this study, it was conjectured that this phenomenon was caused by the low production of VFAs by the *Rikenellaceae RC9 gut group*. Indole alleviates microbial toxicity and plays a crucial role in microbial metabolism and animal health [68]. The tryptophan metabolism pathway constitutes the primary synthetic pathway for indole [69]. Indole-3-acetamide synthesizes indole-3-carboxaldehyde in the presence of a catalyst. It has been reported [70] that Indole-3-carboxaldehyde is a metabolite closely associated with rumen development. It promotes rumen epithelial development by activating the Wnt/β-catenin signaling pathway in the rumen. Based on the findings of the present study, factors that impact rumen microbial composition, rumen metabolite content, fermentation parameters and the activity of digestive enzymes and rumen morphology can be hypothesized (Figure 8). Despite the findings of the present study, the mechanism of rumen microbial composition, rumen metabolites, fermentation parameters and digestive enzyme activity affecting rumen morphology still needs further study.

In this study, it was found that dietary protein levels significantly impacted the biosynthesis of unsaturated fatty acids. A small proportion of UFAs not hydrogenated by microorganisms in the rumen is present in free form [71]. On the one hand, free UFAs can be toxic to microorganisms, which decreases their abundance. On the other hand, free UFAs can bind to fiber in feeds, which inhibits the activities of microbial enzymes that digest fiber. This, in turn, reduces their ability to digest fiber [72,73]. It has been reported that the abundance of *Ruminococcus 1* is associated with the biohydrogenation of rumen fat [74]. In this study, it was found that *Prevotella 1* was the most abundant genus across the three groups, and its abundance was strongly and negatively correlated with stearic and palmitic acids. How *Ruminococcus 1* degrades several substrates and exhibits high resistance to UFAs has been investigated [67]. In the present study, it was found that the abundance of *Ruminococcaceae UCG-014* was significantly lower than that of *Prevotella 1*. The abundance of *Ruminococcaceae UCG-014* was strongly and positively correlated with oleic acid and erucic acid contents, supporting the above analysis. The relationship between melatonin and the abundance/richness of gut microbiota has garnered significant attention in recent years. It has been demonstrated that melatonin impacts microbial abundance [75]. In the present study, the content of 6-hydroxymelatonin was strongly and positively correlated with the abundance of *Ruminococcaceae UCG-014*, *Coprostanoligenes group*, *Ruminococcus 1* and *Succiniclasticum*, suggesting that 6-hydroxymelatonin affects the abundance of rumen flora. Taurine levels increased with the abundance of *Ruminococcus 1* [76]. A strong, positive correlation was also observed between the abundance of *Ruminococcus 1* and taurine content.

## 5. Conclusions

Given the findings that hybridization altered the microbiome (*Prevotella 1*, *Rikenellaceae RC9 gut group*, *Candidatus Saccharimonas*, *Ruminococcus 1*, *Coprostanoligenes group*, *Ruminococcaceae UCG-014*, *Lachnospiraceae NK3A20 group* and *Succiniclasticum*) and metabolome (stearic acid, oleic acid, palmitic acid, erucic acid, 6-hydroxymelatonin, indole-3-acetamide and taurine), it is possible to explain how the high-protein diet (13.03%) affects the fermentation parameter, papilla development and digestive enzyme activity of Tibetan sheep. Taken together, additive inclusion to 13.03% CP diet had a more complementary effect on rumen function compared to 11.58 and 10.20% CP diets, which provided the theoretical basis for formulating a more scientific and reasonable supplementary diet in the future.

## Figures and Tables

**Table 1:** Ingredients and nutrient levels of the diets (dry matter basis), %.

Items	Group		
	HP	MP	LP
Ingredient			
Oat hay	15.00	15.00	15.00
Oat silage	15.00	15.00	15.00
Corn	32.41	32.20	37.10
Wheat	4.90	7.70	7.70
Soybean meal	5.60	1.40	0.70
Rapeseed meal	11.20	11.20	7.00
Cottonseed meal	1.40	0.70	0.70
Maize germ meal	1.40	0.70	0.70
Palm meal	8.40	11.20	11.20
NaCl	0.62	0.68	0.59
Limestone	0.70	0.70	0.70
Baking soda	0.07	0.07	0.07
Premix ^1^	2.94	2.94	2.94
Lysine	0.29	0.39	0.48
Methionine	0.07	0.13	0.11
Total	100.00	100.00	100.00
Nutrient levels ^2^			
Digestibility/MJ·kg^−1^	9.61	9.61	9.62
Crude protein	13.03	11.58	10.20
Ether extract	3.36	3.36	3.36
Neutral detergent fiber	32.95	33.03	32.82
Acid detergent fiber	15.25	15.43	15.04
Calcium	1.10	0.99	0.95
Phosphorus	0.60	0.57	0.55

^1^ The premix provided the following per kg of diets: Cu 18 mg, Fe 66 mg, Zn 30 mg, Mn 48 mg, Se 0.36 mg, I 0.6 mg, Co 0.24 mg, vitamin A 24,000 IU, vitamin D 4 800 IU and vitamin E 48 IU. ^2^ Digestible energy is calculated and the rest are measured. HP, high protein. MP, moderate protein. LP, low protein.

**Table 2:** Fermentation parameter determination.

Item	HP	MP	LP	*p*-Value
pH	6.24 ± 0.06	6.42 ± 0.13	6.67 ± 0.09	0.076
Ammonia nitrogen (mg·L^−1^)	329.53 ± 16.24 ^a^	235.83 ± 17.83 ^b^	225.25 ± 13.58 ^b^	0.001
Total VFAs (mmol·mL^−1^)	131.61 ± 7.23 ^a^	108.21 ± 4.80 ^b^	106.62 ± 1.38 ^b^	0.023
VFA (mmol·mL^−1^)				
Acetate	80.82 ± 2.87	78.79 ± 2.09	80.12 ± 3.41	0.806
Propionate	28.80 ± 0.87 ^a^	23.25 ± 1.25 ^ab^	17.00 ± 1.78 ^b^	0.001
Butyrate	12.77 ± 0.62 ^a^	7.30 ± 0.92 b	9.11 ± 0.81 ^b^	0.001
Isobutyrate	1.95 ± 0.09	1.57 ± 0.11	1.99 ± 0.07	0.058
Valerate	2.32 ± 0.24	2.01 ± 0.12	1.69 ± 0.20	0.056
Isovalerate	4.17 ± 0.37	3.72 ± 0.62	3.95 ± 0.74	0.581

^a,b^ Means with different superscripts in the same row are significantly different (*p* < 0.05). Data are presented as mean ± SEM. The protein level in the HP group was 13.03%, while the protein level in the MP group was 11.58% and in the LP group was 10.20%.

**Table 3:** Determination of digestive enzyme activity.

Item	HP	MP	LP	*p*-Value
α-Amylase (μmol·L^−1^)	194.52 ± 3.33 ^a^	183.45 ± 11.29 ^ab^	144.01 ± 4.38 ^b^	0.001
Trypsin (ng·mL^−1^)	490.10 ± 16.41 ^a^	442.26 ± 11.94 ^b^	446.67 ± 13.42 ^b^	0.039
Cellulase (ng·L^−1^)	153.61 ± 13.89	140.64 ± 9.66	138.98 ± 5.96	0.542
Lipase (ng·L^−1^)	471.09 ± 16.41	445.67 ± 21.94	446.48 ± 13.42	0.139

^a,b^ Means with different superscripts in the same row are significantly different (*p* < 0.05). Data are presented as mean ± SEM. The protein level in the HP group was 13.03%, while the protein level in the MP group was 11.58% and in the LP group was 10.20%.

## Data Availability

The datasets presented in this study can be found in online repositories. The names of the repository/repositories and accession number(s) can be found below: NCBI SRA (accession: PRJNA1095164). MetaboLights (MTBLS10452. https://www.ebi.ac.uk/metabolights/editor/study/MTBLS10452, accessed on 16 June 2024).

## Supplementary Materials

The following supporting information can be downloaded at: https://www.mdpi.com/article/10.3390/ani14152149/s1, Figure S1: Venn diagram of ruminal microbial diversity; Figure S2: The values of correlation coefficients between the QC samples; Figure S3: Superclass of metabolites; Figure S4: Volcano diagram of three groups of metabolites.
