# Supplementary figures and images for: Integrating 16S rRNA Sequencing and LC-MS-Based Metabolomics to Evaluate the Effects of Dietary Crude Protein on Ruminal Morphology, Fermentation Parameter and Digestive Enzyme Activity in Tibetan Sheep
Source: Animals (Basel). 2024 Jul 24;14(15):2149. doi: 10.3390/ani14152149 (PMC11310993; doi:10.3390/ani14152149)

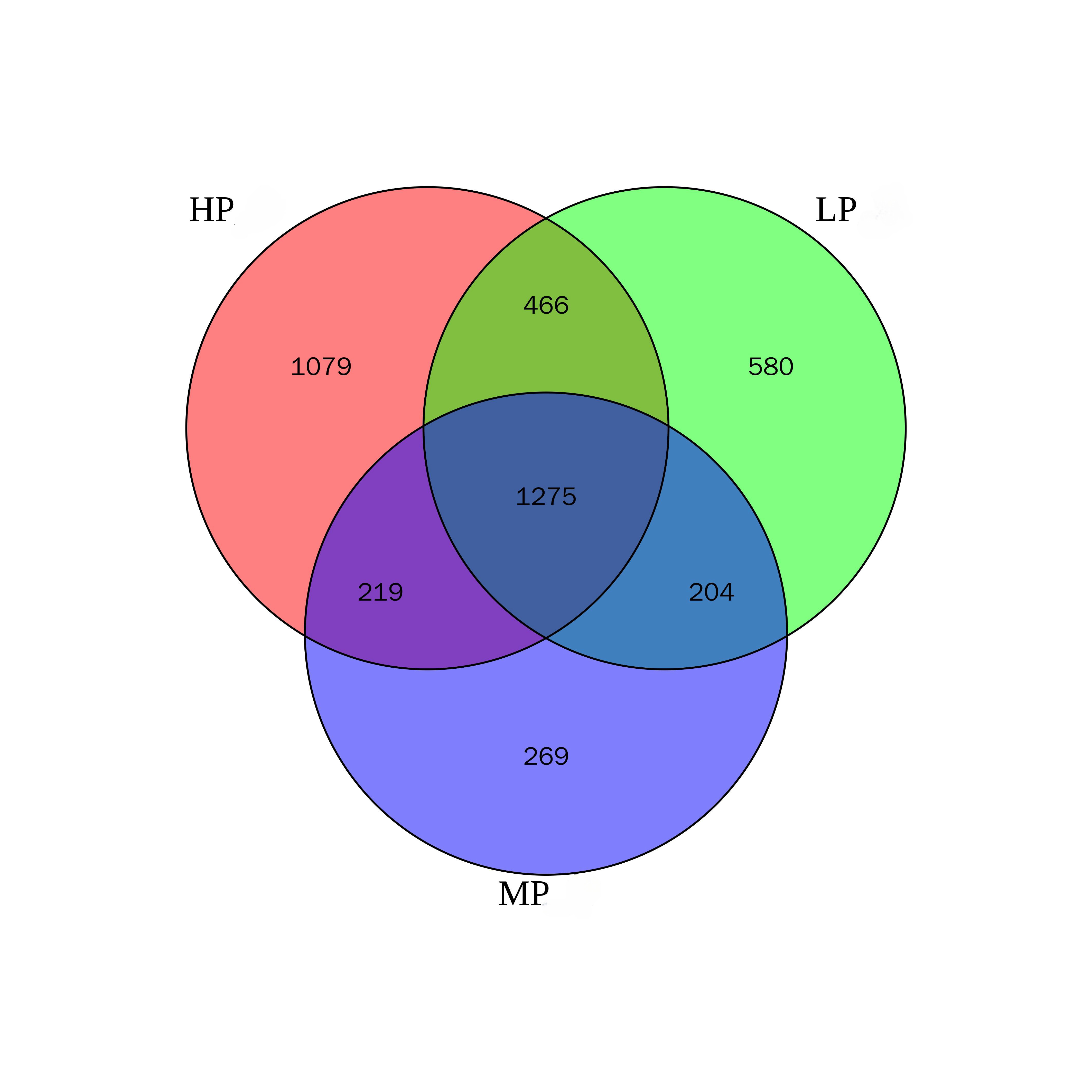

Supplement: Supplementary file 1 [file animals-14-02149-s001.zip › Figure S1.TIF]

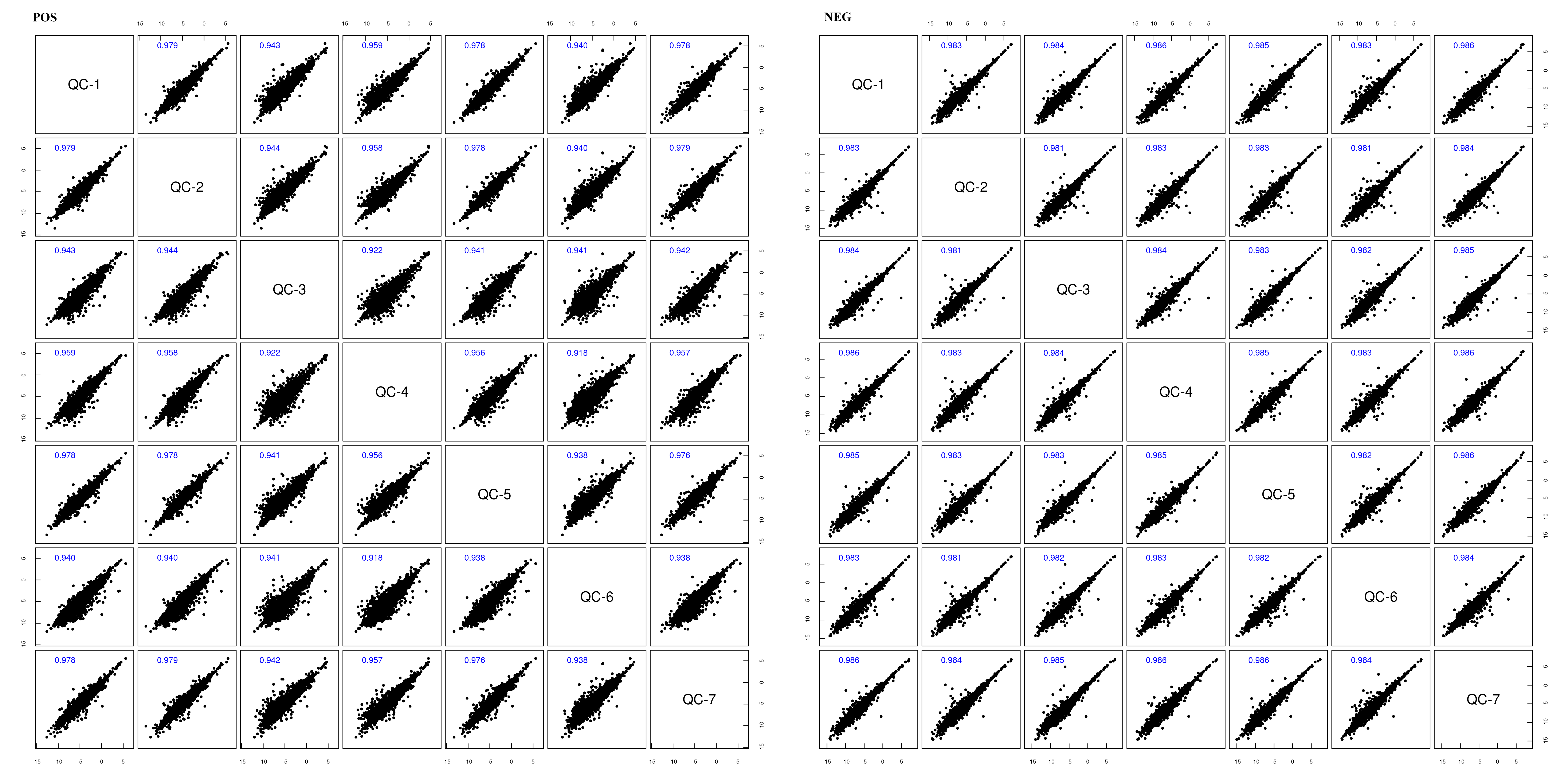

Supplement: Supplementary file 1 [file animals-14-02149-s001.zip › Figure S2.TIF]

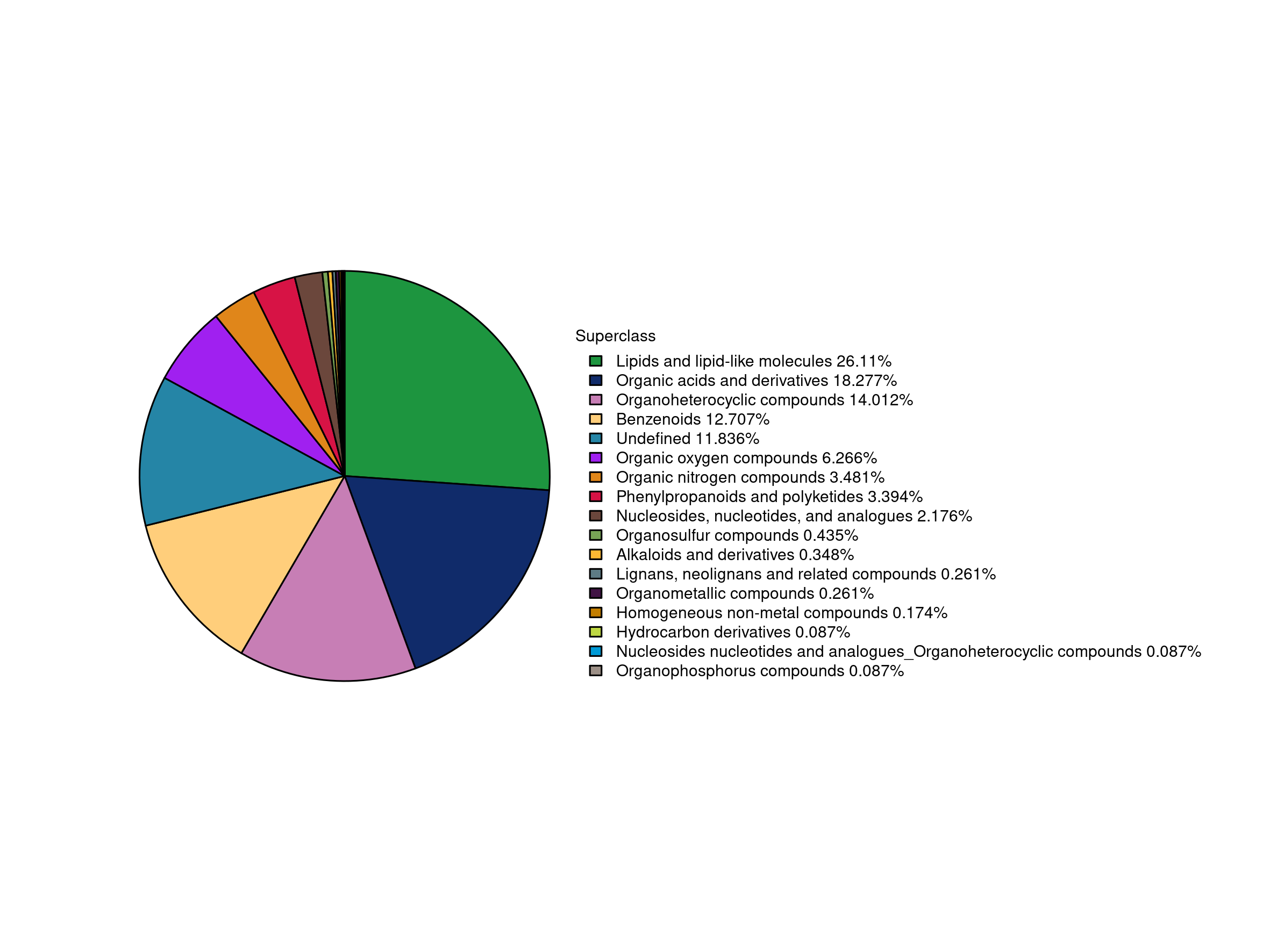

Supplement: Supplementary file 1 [file animals-14-02149-s001.zip › Figure S3.TIF]

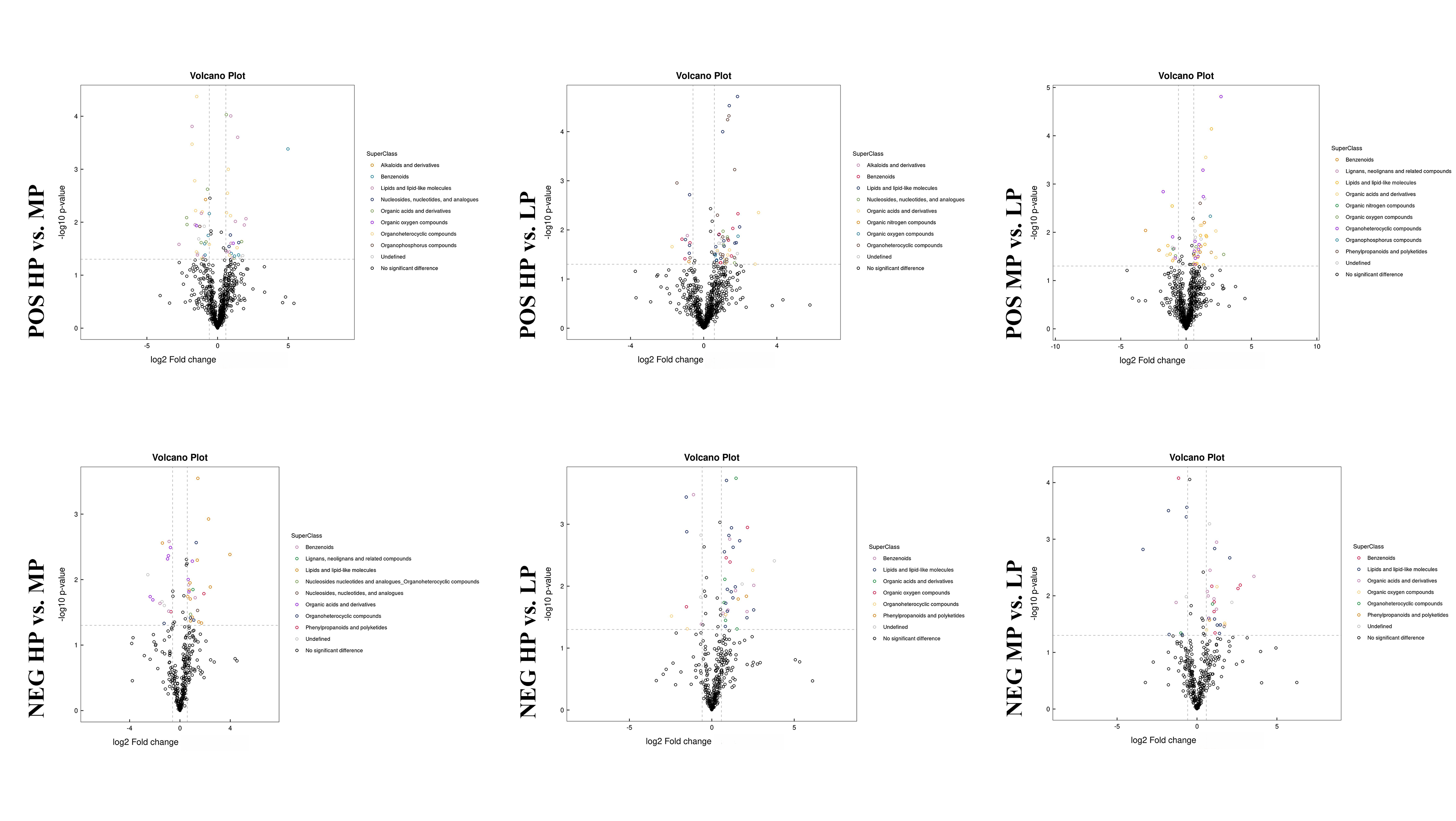

Supplement: Supplementary file 1 [file animals-14-02149-s001.zip › Figure S4.TIF]
